# Evolutionary mechanisms modulating the mammalian skull development

**DOI:** 10.1098/rstb.2022.0080

**Published:** 2023-07-03

**Authors:** Stella Kyomen, Andrea P. Murillo-Rincón, Markéta Kaucká

**Affiliations:** Max Planck Institute for Evolutionary Biology, August-Thienemann-Strasse 2, Plön 24306, Germany

**Keywords:** mammalian skull evolution, craniofacial development, heterochrony, heterotopy and heterometry, morphological variation

## Abstract

Mammals possess impressive craniofacial variation that mirrors their adaptation to diverse ecological niches, feeding behaviour, physiology and overall lifestyle. The spectrum of craniofacial geometries is established mainly during embryonic development. The formation of the head represents a sequence of events regulated on genomic, molecular, cellular and tissue level, with each step taking place under tight spatio-temporal control. Even minor variations in timing, position or concentration of the molecular drivers and the resulting events can affect the final shape, size and position of the skeletal elements and the geometry of the head. Our knowledge of craniofacial development increased substantially in the last decades, mainly due to research using conventional vertebrate model organisms. However, how developmental differences in head formation arise specifically within mammals remains largely unexplored. This review highlights three evolutionary mechanisms acknowledged to modify ontogenesis: heterochrony, heterotopy and heterometry. We present recent research that links changes in developmental timing, spatial organization or gene expression levels to the acquisition of species-specific skull morphologies. We highlight how these evolutionary modifications occur on the level of the genes, molecules and cellular processes, and alter conserved developmental programmes to generate a broad spectrum of skull shapes characteristic of the class Mammalia.

This article is part of the theme issue ‘The mammalian skull: development, structure and function’.

## 1. Introduction

The mammalian lineage evolved from reptilian forebears around 178 Ma during the Mesozoic era [1]. Modern mammals, traditionally divided into monotremes, marsupials and placentals, comprise over 6000 species that occupy an immense variety of ecological niches [2]. The evolution of multiple novel traits and the morphological diversity, especially within the craniofacial region, enabled the adaptations to life in various environments [3]. The head hosts tissues and organs that fulfil an array of functions essential to life. The fundamental role in tissue and organ integration is attributed to the bony skull, which also determines the general shape of the head. The skull is essential for protecting sensitive sensory organs such as the eyes, the olfactory system, the inner ear and the central nervous system. Additionally, the skull and its specific shape dictate the position of structures within the head and provide attachment points for the muscles. Therefore, skull shape diversity enabled mammals to develop diverse modes of food acquisition and processing, orientation in the environment, communication and physiological functions.

The morphological innovations and skull shape variability in mammals are ultimately reflections of changes in embryonic development during evolution. The development of the head is a multistep process orchestrated by developmental signalling pathways that are conserved across vertebrates [4,5]. How a defined set of conserved genes generates fundamentally different skull shapes has been of interest for the last decades, and our knowledge has grown considerably thanks to extensive research using conventional vertebrate models such as zebrafish, *Xenopus*, chick and mouse [6–9]. Traditional approaches in developmental biology, comparative morphology, quantitative morphometrics, live imaging and transgenesis have provided us with a solid comprehension of the discrete yet intertwined developmental events and the underlying molecular gears operating during vertebrate head morphogenesis. Mammalian craniofacial research has mainly relied upon the mouse model due to its amenability to genetic perturbations and lineage tracing, and its fast reproduction rate. However, mammals and their extraordinary natural craniofacial variability represent an exceptional model for studying the evolution of developmental processes. Therefore, comparative research of mammalian craniofacial development across taxa will be instrumental in comprehending how morphological variation arises during the embryogenesis of closely related species.

Patterns of morphological and transcriptome profile conservation and divergence along the ontogeny have been observed across vertebrates. The hourglass model of embryonic evolution proposes that the mid-embryonic stages, known as a phylotypic period, evince morphological and transcriptional conservation, while the early and late embryonic stages are divergent across species [10–15]. The phylotypic period is acknowledged as the source of the general body plan, and the increasing divergence after this stage accounts for the formation of species-specific characteristics. Several mechanisms modulating the developmental processes and thus contributing to the increasing morphological divergence along the ontogeny were identified. Among them, heterochrony, heterotopy and heterometry were most extensively investigated, and their effects are tightly intertwined [16,17]. In the following sections, we present how these mechanisms of evolutionary change substantially alter or fine-tune conserved developmental processes and allow the formation of a broad spectrum of craniofacial shapes. We want to emphasize that the modulations occur on numerous levels, such as gene expression, molecule activity and stability, cell fate acquisition, cell behaviour and polarity, tissue interactions and patterning. Each such modification will subsequently impact the following developmental steps and ultimately establish the unique geometry of individual craniofacial structures or the whole head. In this review, studies of developmental modifications that emerged naturally are primarily presented; however, we also complement the evidence with research using model organisms and genetic perturbations.

## 2. Key evolutionary mechanisms modulating ontogenesis

The definition of heterochrony historically changed multiple times. The original view described heterochrony as a change in the timing of feature appearance in the sequence of developmental events between an ancestor and its descendant. Decades later, the term heterochrony was used to describe relative changes in the timing, relative rate or duration of developmental events between related species that generate morphological diversity [16,17]. The current view perceives heterochrony as a genetically driven modulation of timing, rate or duration that appears on distinct levels, for instance, molecular, cellular or morphological [16,18,19]. Particularly, heterochrony in gene expression controlled by *cis*-regulatory elements has been in the spotlight in recent years and is acknowledged to be a major source of morphological variability [20,21].

Another mechanism of evolutionary change is heterotopy, which generally denotes a change in spatial arrangements. Similar to heterochrony, the classical emphasis of heterotopy was narrower and referred to changes in germ layer origin. Later, the term heterotopy was used to denote the distinct (positional) origin of precursors forming homologous structures, the altered spatial organization of dynamic processes such as proliferation in the growth zones or a positional change of a morphological structure relative to the ancestor [16,22]. However, the modern definition of heterotopy also recognizes changes in the spatial patterns of gene expression, enhancer activity, protein distribution, cell behaviour (e.g*.* intensity or polarity of cell proliferation) and internal architecture [16,22,23]. Molecular heterotopy also received renewed attention in the last two decades, where differential gene expression patterns were associated with the formation of distinct morphologies found in vertebrates. Molecular heterotopy often triggers subsequent changes, for instance, of the tissue patterning or the size, shape or position of zones displaying specific cell behaviours (e.g. cell migration or division). These altered processes are further potent modulators of tissue morphogenesis and significantly contribute to the acquisition of the species-specific head and skull geometry [24,25].

The third mechanism modulating developmental events is heterometry, which initially denoted changes in the number of morphological features [16]. However, nowadays, the term also encompasses molecular heterometry, referring to quantitative changes in gene expression levels and protein concentration. The best-studied effect of molecular heterometry is the case of secreted ligands that affect morphogenesis in a concentration-dependent manner and are known to underlie substantial changes in craniofacial morphology [26–28]. A study comparing the development of two amphibian species (*Xenopus laevis* and *Xenopus tropicalis*) suggested that heterometry of gene expression is more frequent than molecular heterochrony in closely related species [29].

These three evolutionary mechanisms have been explored mainly across larger evolutionary scales and are proposed to underlie the origin of novel traits and the morphological variation in vertebrates [30–33]. However, to fully comprehend the contribution of heterochrony, heterotopy and heterometry to morphological diversity in mammals, there is a need for comparative studies that will investigate discrete molecular, cellular and developmental processes together with the underlying genetic background, across species and along the relevant developmental timeline. In the following section, we present selected studies demonstrating specific cases of ontogenetic modifications and their effects on skull formation and shaping in diverse mammalian species. We intend to highlight that these modifications can occur at any developmental stage and on molecular to tissue levels, thus representing an enormously flexible system to alter craniofacial morphology with only moderate changes in genetic information. The presented research also pinpoints the need to understand which is the causative modulation and how it modifies the consecutive steps of head morphogenesis.

## 3. Heterochrony, heterotopy and heterometry in mammalian craniofacial development

**Figure 1.**
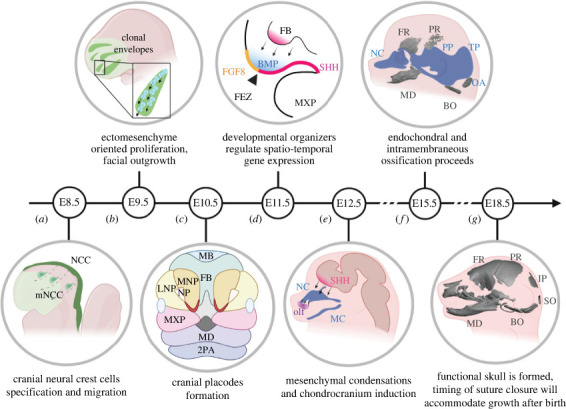
Timeline of craniofacial development in the mouse model. (*a*) Embryonic day (E) 8.5. Cranial neural crest cells delaminate and migrate from the borders of the closing neural tube to the presumptive head. (*b*) At E9.5, the CNCC-derived ectomesenchyme undergoes high proliferation, generating clones that spatially overlap and forming facial prominences. (*c*) At E10.5, the facial prominences continue to grow, and the olfactory pits and mediolateral processes develop. (*d*) At E11.5, the FEZ developmental organizer is defined by the mutually exclusive expression of *Shh* and *Fgf8*, and regulates vertebrate facial morphogenesis. (*e*) At E12.5, the mesenchymal condensations form in the face, and their geometry represents the original blueprint of the skull shape. (*f*) At E15.5, endochondral and intramembranous ossification proceeds. The chondrocranium grows extensively, and the ossification centres of the cranial vault are formed. (*g*) E18.5. A functional skull is formed and the patent cranial sutures accommodate the brain's and skull's postnatal growth. NCC, neural crest cells; mNCC, migratory neural crest cells; olf, olfactory placode; FGF8, fibroblast growth factor 8; BMP, bone morphogenic protein; SHH, sonic hedgehog; MD, mandibular prominence; MXP, maxillary prominence; LNP, lateral nasal prominence; MNP, medial nasal prominence; NP, nasal pit; FB, forebrain; MB, midbrain; 2PA, second pharyngeal arch; FEZ, frontonasal ectodermal zone; NC, nasal capsule; MD, mandibular bone; MC, Meckel's cartilage; PP, parietal plate; TP, tectum posterius; OA, occipital arch; FR, frontal; PR, parietal; BO, basioccipital; IP, interparietal; SO, supraoccipital. In (*e*,*f*,*g*) blue represents cartilage and grey shows bone.

The embryonic formation of the head is a sequence of tightly connected events. Cells from all germ layer origins contribute to head formation, and their behaviour, such as proliferation, migration or cell fate acquisition, is coordinated at a genomic, molecular and cellular level (figure 1). Although conserved molecular programmes drive head morphogenesis in all vertebrates, differences in their utilization result in the emergence of inter- and intra-species differences. A vast amount of research has been conducted using vertebrate models such as zebrafish, chicken or *Xenopus* due to their accessibility and the possibility of observing their development *in vitro* or *in ovo*. A large proportion of obtained knowledge can be extrapolated to mammals. However, the developmental differences that give rise to mammalian-specific features and govern the acquisition of species-specific geometries have not yet been systematically investigated. In the following sections, we present selected studies that researched diverse mammalian species and noted developmental differences generating morphological variability. We set out to highlight how distinct craniofacial mammalian features and geometries arise from modulations of conserved molecular programmes without an extensive change of coding genetic information.

### (a) The cranial neural crest cells

The neural crest is a multipotent embryonic population conventionally divided into the cranial, cardiac, vagal, trunk and sacral, based on their position along the anteroposterior axis of the embryo [34]. The cranial neural crest cells (CNCCs) are formed within the most anterior domain of the developing neural tube and are essential for vertebrate craniofacial development [35–37]. CNCCs give rise to a repertoire of cell types and tissues, such as the craniofacial skeleton, connective tissues, glial cells, melanocytes, peripheral sensory neurons, parasympathetic and sympathetic neurons, adipocytes and others. The major events in the development of the CNCCs are conserved across vertebrates, although spatio-temporal differences in gene expression patterns and CNCCs behaviour are found across species [38,39].

Neural crest induction occurs during gastrulation and is initiated by the expression of the neural plate and neural border specifiers such as *Pax 3/7*, *Snai2*, *Msx1/2* and *Zic1* [40,41]. The next step in the CNCCs emergence is the activation of neural crest specifier genes such as *Sox9* and *Sox10* [40–42]. During neurulation (folding and closure of the neural plate to form the neural tube), premigratory CNCCs undergo epithelial-to-mesenchymal transition (EMT), which allows them to delaminate from the neural tube and migrate along conserved migratory paths [39,43]. The timing of EMT, delamination and migration varies across vertebrates, and this heterochrony is likely related to the differential expression of cell adhesion and cell polarity genes that control this process. Particularly in mammals, these three events occur shortly after one another [44–46]. CNCCs subsequently migrate to their final destinations in the forming head, giving rise to a broad spectrum of derivatives (figure 1*a*) [4,24].

**Figure 2.**
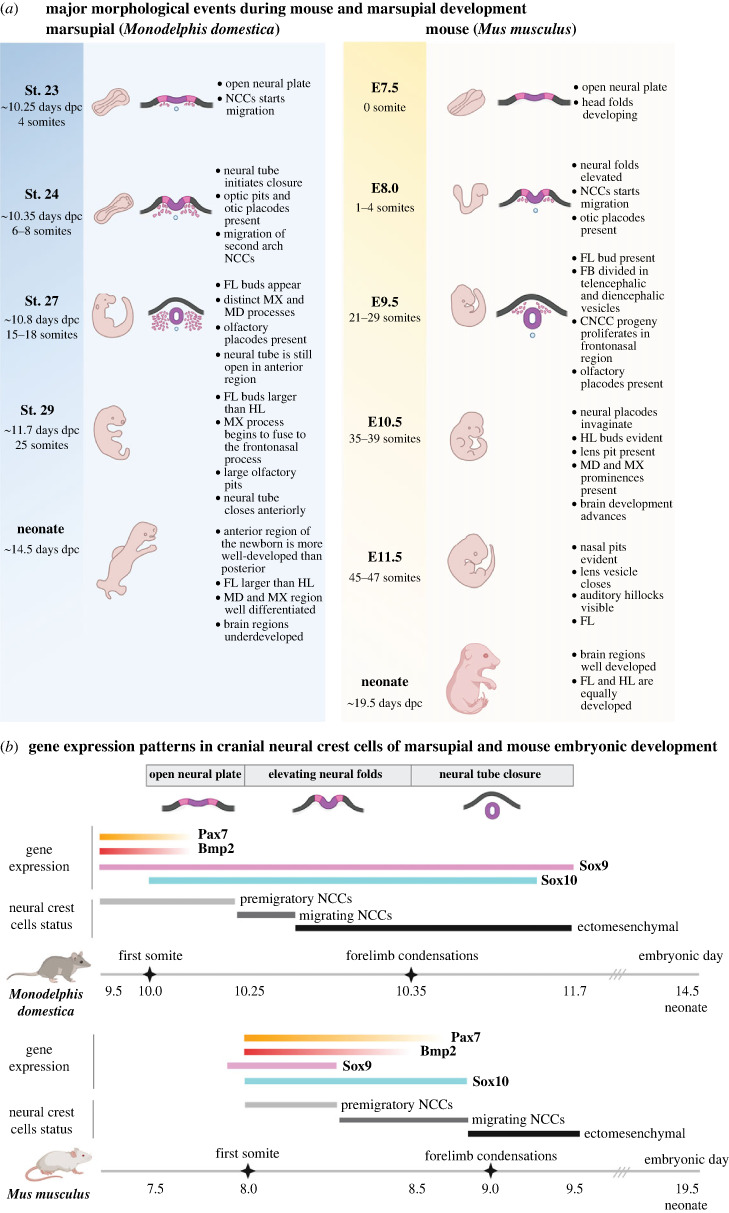
Neural crest specification heterochrony in marsupials. (*a*) Timeline of the morphological landmark appearance during embryogenesis of marsupial (*Monodelphis domestica*, left) and mouse (*Mus musculus*, right). Marsupial embryos are born at around 14.5 days of gestation and morphologically resemble E11.5 mouse embryo. Heterochronic shifts in craniofacial development in marsupials involve delayed differentiation of the central nervous system and accelerated musculoskeletal development in the orofacial and forelimb regions. (*b*) Schematic timeline of gene expression pattern and heterochrony in marsupial and mouse CNCC development. In *M. domestica*, CNCC-specifiers *Sox9* and *Sox10* are expressed earlier (at the open neural plate stage) and longer (until E11.7) compared to a mouse. In a mouse model, the onset of *Sox9* and *Sox10* expression occurs at the neural fold stage, and the *Sox9* expression ceases with the onset of neural crest migration. In the marsupial, the expression pattern of a neural border specifier *Pax7* spans the neural plate stage (stage 20, at around E9.5), while in mouse, the onset of *Pax7* occurs when the neural folds elevate. CNCC formation in marsupial embryos relies on the expression of *Bmp2* in the early developmental stages (E9.0–10.25). Note the heterochronic shift of somitogenesis and forelimb bud development in *M. domestica.* Modified from [40,42]. NCC, neural crest cell; CNCC, cranial neural crest cell; FL, forelimb; HL, hindlimb, MX, maxillary; MD, mandibular.

Despite the limited cross-species comparative information about the course of CNCC specification and migration in mammals, the existing evidence highlights the impact of heterochrony in CNCC development and its subsequent effects on skull formation and species-specific ecological requirements. The best example of this impact is the heterochrony in CNCC specification and migration observed in the grey short-tailed opossum (*Monodelphis domestica*), a representative of marsupials. Compared to placental mammals, CNCC specification and migration occur significantly earlier in marsupials, even before somite and brain patterning are evident (figure 2*a*) [47–49]. This heterochronic shift in CNCC specification and migration in the opossum is associated with the differential spatio-temporal expression of *Pax7* and *Sox9* (figure 2*b*) [40]. The onset of *Sox9* expression in marsupials occurs earlier than in the mouse (i.e. prior to neural fold elevation) and is controlled by a marsupial-specific *Sox9* enhancer [40]. The activity of the Sox9 enhancer is additionally associated with the extended duration of *Sox9* expression in the facial ectomesenchyme in the opossum, while in the mouse, *Sox9* expression ceases after CNCCs emigrate to the arising face. The molecular heterochronic shift of *Sox9* expression causes the early onset of CNCC specification and migration and results in the early formation of jaw primordia relative to other structures, such as the brain that remains underdeveloped compared to mouse embryos of similar developmental stage [48]. Consequently, the head of marsupial neonates resembles a mouse embryo at approximately 11.5 days after fertilization (E11.5). While marsupial newborns possess poorly developed brains and cranial bones, the oral region and the forelimbs are strikingly well-developed and functional [19,23,50]. This phenomenon is in accord with the functional demands faced by marsupial neonates that are altricial at birth and need functional forelimbs to reach the mother's pouch and efficient feeding apparatus to suckle milk.

### (b) The ectomesenchyme and the mesenchymal condensation

Once the CNCCs reach their final destination, migration ceases, and the cells undergo extensive proliferation to form the facial ectomesenchyme. The ectomesenchyme primarily occupies the anterior part of the head, while mesoderm-derived mesenchyme mainly forms the posterior part [24,51–53]. However, there is a certain level of mixing between the mesenchyme of both origins [4,24,54].

**Figure 3.**
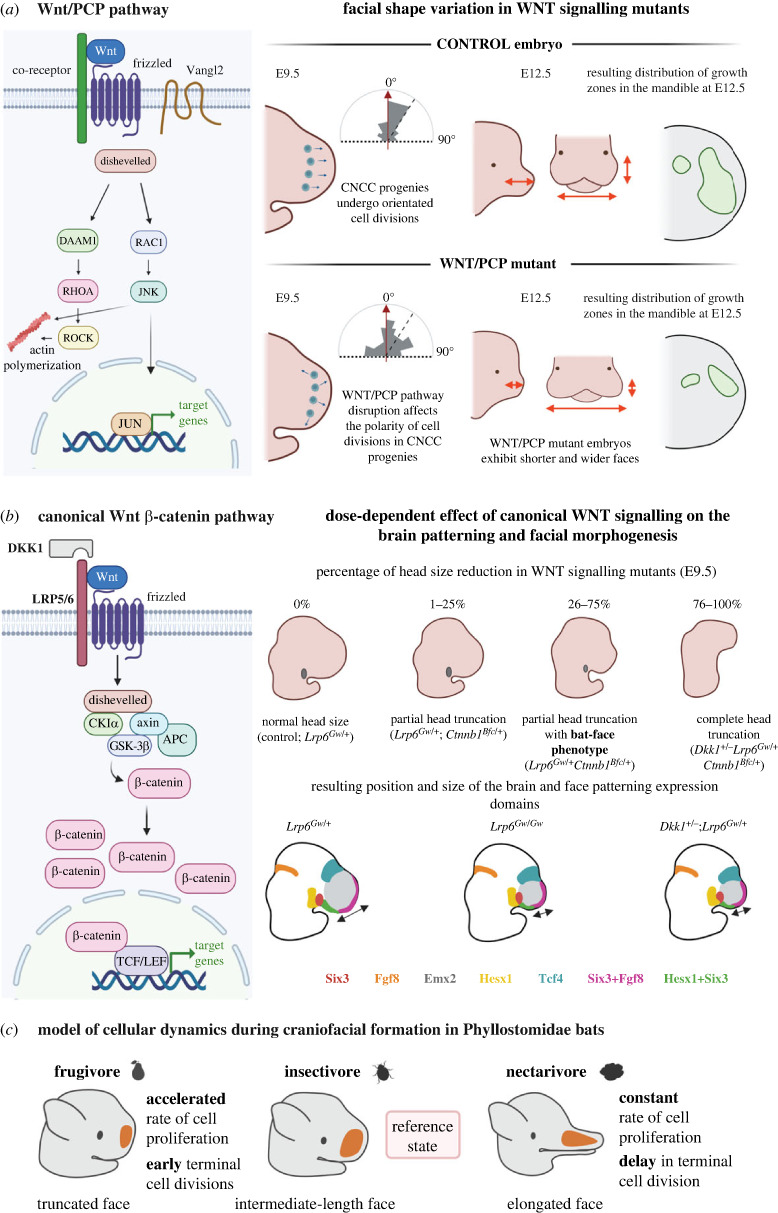
Facial shape variation generated by heterometry of WNT signalling components. Schematic illustration of the non-canonical WNT/PCP pathway that controls the directionality of cell divisions in CNCC-derived ectomesenchyme during mouse embryogenesis. Analysis of the cell division polarity (rose diagrams) in the snout shows disrupted directionality in WNT/PCP knockout embryos. Craniofacial morphology in control and WNT/PCP mutants differ dramatically, and mutant embryos show wider and shorter facial proportions. Note the different distribution and size of proliferative zones in E12.5 mandible between controls and mutants. Adapted from [24]. (*a*) Schematic illustration of canonical WNT (β-catenin dependent) pathway. Mutagenesis in a mouse model targets LRP5/6 co-receptors, DKK1 secreted antagonist and β-catenin transcriptional co-activator. Brain patterning and facial morphogenesis are strongly affected by the dose of the pathway components and various levels of head reduction were observed in E9.5 mutant embryos. The genetic perturbation caused the activation of the canonical WNT pathway in a much larger area of the developing head and altered the brain and facial morphogenesis. The resulting bat-face-like phenotype with truncated snout was observed in a proportion of *Lrp6^Gw/+^Ctnnb1^Bfc/+^* mutants. Note that the heterometric change of canonical WNT signalling in the mutant embryos altered the facial proportions early in development. The general positioning of the consecutive developmental organizers remained similar to the controls, but the size of the expression zones in mutants was affected. Adapted from [60]. (*c*) Cell proliferation model of facial development in Phyllostomidae bats. Heterochronic and heterotopic modulation of cell proliferation zones underlies snout length variation in Phyllostomidae bats. Interspecific differences may arise from altered cellular proliferation during mesenchymal condensation or cartilage growth or by the shift of the ossification onset. Frugivore species possessing truncated facial proportions possess a high proliferation rate in the midfacial region. The elongated facial region of nectarivore bats is associated with a constant cell proliferation rate and a delay in ossification. Adapted from [32,61].

Anteriorly, the ectomesenchyme gives rise to the facial prominences that fuse later in development to form the upper face [55], the cranial base and several bones of the cranial vault [52,53,56]. Notably, the polarity of cell division during ectomesenchyme proliferation is essential for establishing the general geometry and outgrowth of the face [24,57–59]. Functional experiments using a mouse model pinpointed the essential role of the non-canonical WNT/PCP (Wingless-Integrated, Planar Cell Polarity) pathway in establishing the polarity of the facial ectomesenchyme and thus controlling face shaping (figure 3*a*). Mutation of Wnt5a or Ror2/Vangl2, the components of the WNT/PCP pathway, resulted in the formation of brachycephalic geometries characterized by shorter and wider facial proportions [24,57,58]. The extent of cell proliferation in the mutant embryos remained comparable to the wild-type. However, the random directionality of daughter cell allocation in the fast-proliferating ectomesenchyme of WNT/PCP mutants affected the facial outgrowth and gave rise to brachycephalic facial geometry. Consequent heterotopic changes progressively occurred and affected, for instance, the size and shape of growth zones that dictate the further outgrowth of the face [24].

Genome-wide association studies (GWAS) on dozens of dog breeds identified genomic variants in WNT signalling pathway components, specifically in breeds with brachycephalic appearance, widely positioned eyes and shorter statues [62]. For instance, the facial morphology of dog breeds with identified frameshift mutation of *Dvl2*, a mediator of both canonical and non-canonical WNT signalling, resembles the phenotype of mice with a genetically perturbed WNT pathway [57,60,62]. Similarly, genetic perturbations of canonical WNT components such as Dkk1, β-catenin or LRP6 resulted in the ectopic activation of canonical WNT signalling in the developing face, where embryos recapitulate the phenotype of WNT/PCP mouse mutants and, according to the authors, strikingly resemble the short snout of fruit-eating bats (figure 3*b*) [60].

Interestingly, shorter facial proportion or reduced projection of the muzzle, the semiflexible mammalian tip of the snout, are generally associated with domestication (e.g. pigs and goats) or breed production (e.g. dogs and cats), although this phenotype also occurs naturally (e.g. orangutans and some bats) 63]. This observation led to the ‘neural crest/domestication syndrome’ hypothesis that linked the phenotypic hallmarks of domestication to the biology of the NCCs [64,65]. However, this hypothesis has neither been reliably confirmed nor widely accepted [66,67]. Systematic research on cell polarity factors and ectomesenchyme behaviour (i.e. extent and directionality of cell divisions) in wild and domesticated animals with brachycephalic facial proportions has not been carried out yet. However, it is possible to speculate that the distinctive brachycephalic geometry of numerous domesticated animals may be related to heterotopic and heterometric changes in the expression and activity of the WNT pathway during early facial formation, and specifically affecting ectomesenchyme proliferation and leading to downstream changes in the facial growth.

Other candidate genes can be inferred from the GWAS studies on brachycephalic dog breeds, for instance, *Bmp3* and *Smoc2* [68,69]*.* These two genes are tightly associated with craniofacial development. Brachycephalic dogs possess a *Bmp3* variant that is conserved in all breeds examined. This particular variant is believed to be inactivating, resulting in a reduced *Bmp3* dose (heterometric change) in these animals. In the mouse, *Bmp3* is reportedly involved in endochondral bone formation [70]. However, although *Bmp3* is also expressed in craniofacial bones and mesenchyme [71], the *Bmp3* knockout mouse model does not manifest any craniofacial abnormality [72]. Contrary to that, blocking the translation of Bmp3 in zebrafish confirmed its essential role in craniofacial morphogenesis [68]. The *Smoc2* knockout mouse model mimics the craniofacial features observed in brachycephalic dogs [69]. Interestingly, deleterious mutations in this gene were also found in human patients with craniofacial and dental malformations [73]. Altogether, the evidence from brachycephalic species and genetic perturbations in the mouse model jointly highlight the importance of gene dose (heterometry) for craniofacial formation and accentuates the cascade of further (heterometric and heterotopic) changes caused by this initial modulation. Thus, cell polarity pathways and ectomesenchyme behaviour may represent the prime targets for investigating developmental differences in species with shorter and wider facial proportions.

After the formation and outgrowth of the ectomesenchyme, intense proliferation in specific areas of the facial mesenchyme results in its compaction and the formation of mesenchymal condensations [74]. Mesenchymal condensations differentiate into the cartilage or bone shortly after their formation [74–77]. Whether the condensation is chondrogenic (forming the cartilage) or osteogenic (differentiating into bone) depends on the bone-forming mechanism of that particular skeletal element, which is classified as endochondral or intramembranous ossification [78]. Notably, the spatial pattern of the newly formed condensations already resembles the general blueprint of the future skeletal elements and thus dictates the number and position of the skeletal elements of the emerging skull [57,77].

How exactly the mesenchymal condensations are induced is not well known yet; however, conserved secreted morphogens from the BMP (bone morphogenetic protein), WNT, FGF (fibroblast growth factor) and Hedgehog families are reportedly directly and indirectly involved in mesenchyme patterning, induction of mesenchymal condensation and chondrogenic or osteogenic differentiation [79–85]. Thus, heterometric and heterotopic changes of these ligands may significantly contribute to mammalian craniofacial variability.

### (c) Developmental organizers

A prominent function in the process of head morphogenesis is attributed to developmental organizers. This term refers to spatially defined areas that release instructive signals, known as morphogens, that provide the neighbouring tissues with a roadmap for further development. Differential (spatio-temporal and quantitative) expression patterns of morphogens are found across vertebrates and are acknowledged to control the establishment of species-specific morphology [79,86–89]. Morphogens such BMPs, WNTs, FGFs and Sonic hedgehog (Shh) are released by distinct organizers throughout embryonic development and instruct craniofacial morphogenesis in a concentration-dependent manner [90–92]. The morphogen expression patterns are dynamically changing in space, time and concentration, and often, the emergence of an organizer is governed by the sequence of instructive events in the earlier stages. Additionally, the morphogens and their downstream effectors frequently form signalling loops among each other; thus, the final morphogenetic event results from the joint action of more than one organizer.

To illustrate the broad spectrum of effects induced by a single morphogen, we would like to describe the example of Shh. The levels of Shh during embryogenesis are controlled by multiple stage- and tissue-specific enhancers that provide for flexible fine-tuning of the expression sites, time or concentration. The reduction of the *Shh* expression in the brain, executed either by tissue-specific ablation of *Shh* or by manipulating the activity of its enhancers, results in a wide range of craniofacial shapes in a mouse model [79,93,94]. Moreover, it is widely recognized that severely reduced and increased levels of *Shh* are the underlying cause of holoprosencephaly (incomplete cleavage of the forebrain) and hypertelorism (widening of the face), respectively [26].

With regard to facial shape acquisition, *Shh* is involved in the establishment of an important facial organizer, the frontonasal ectodermal zone (FEZ) (figure 1*d*) [95]. The FEZ is located in the facial surface ectoderm and is characterized by regions with a mutually exclusive expression of *Shh* and *Fgf8.* FEZ formation requires the Shh signal released from the forebrain in the previous developmental stages [96,97]. The FEZ organizer further requires WNT signalling from the facial ectoderm [98,99] and BMP signalling from the underlying facial mesenchyme [100,101] that together with Shh mediate communication between the frontal facial tissues. The expression pattern of *Shh* and *Fgf8* in the FEZ is known to be species-specific and orchestrates the formation of typical facial features of birds and mammals [87,95,100]. Experimental manipulation of *Shh* and *Fgf8* domains within FEZ in the mouse model to imitate the expression observed in chicken results in a mouse face with chicken-like characteristics. A similar phenotype was obtained by downregulating WNT signalling in the mouse facial ectoderm and interfering with the structure of FEZ and mesenchyme proliferation, giving rise to mouse embryos with a bird-like FEZ and a bird-like narrow, pointy face [99]. Likewise, experimentally induced high expression levels of *Shh* in the chicken forebrain results in a mouse-like FEZ organization and a broader and shorter upper jaw, resembling the facial features of a mouse [102]. Moreover, *Bmp4* expression in the underlying facial mesenchyme also acquires species-specific pattern and controls facial outgrowth and shaping in vertebrates [25,89,103].

Supporting the hypothesis that species-specific expression patterns of morphogens indeed control the acquisition of the specific morphology, experimental manipulation of the BMP signalling cascade produces striking changes in facial shape that resemble naturally occurring phenotypes. Increased *Bmp4* expression in the CNCC-derived ectomesenchyme produces phenotypes resembling certain human facial features in the mouse model: a shorter and narrower face, rounded skull and more anterior orientation of the eyes [104]. Higher *Bmp4* expression was reported in humans compared to their closest relative, the chimpanzee, and this difference is believed to provide for the formation of human facial features [105]. Upregulation of the BMP signalling in the NCCs, executed by genetic ablation of BMP inhibitors, results in the elongation of the face in a mouse model [106,107]. Elevated BMP signalling is observed in the developing face of long-faced bats compared to short, wide-faced bats. The levels of BMP signalling control the establishment of species-specific cell proliferation zones (and cell division rates) that underlie the formation of characteristic phenotypes in these two bat species [107].

Altogether, studies involving genetic perturbations in the mouse model and the observed species-specific patterns provide further evidence for the prominent effect of heterotopy, heterochrony and heterometry of known developmental organizers. This evidence demonstrates that a wide range of morphologies can be generated by altering the spatio-temporal patterns or concentration levels of a few molecules. Interestingly, developmental organizers are often located in developing nervous structures*,* such as the brain and the olfactory epithelium, supporting the hypothesis of co-development and co-evolution of the nervous system and skull [79,108,109]. These observations render the developmental organizers within the nervous system prime targets of evolutionary change and one of the critical drivers of the morphological evolution of the skull.

### (d) Skeletogenesis

Chondrogenesis and ossification represent subsequent developmental events that can be modulated by heterotopy, heterochrony and heterometry to shape the skull. Although bone forms the mammalian postnatal skull, cartilage is fundamental for bone formation as it serves as a template for endochondral and a scaffold for intramembranous ossification in the embryo [110–113]. Cartilage growth during embryogenesis relies upon the proliferation of chondrocytes and perichondrial cells. Heterochrony in cartilage growth is likely to produce notable changes to the final skull shape. For instance, in cetaceans, the striking cranial bone rearrangement repositions the bony nares (the blowhole) dorsally, in a process known as telescoping [114,115], and is associated with heterochronic growth of different parts of the skull. Telescoping involves the elongation of the facial bones (maxilla and premaxilla), the reduction or loss of the nasal bones, and the emergence of ‘horizontal’ sutures between overlapping cranial bones, a process that takes place during embryonic development [116]. This bone rearrangement gives the skull its characteristic elongated and flattened shape. In Odontocetes (toothed whales), telescoping involves the heterochronic shift of formation and outgrowth of the nasal cartilages, which has been proposed to facilitate echolocation [114]. In some Odontocetes, the length of the nasal cartilage, which can exceed three times the width of the skull, is associated with its accelerated growth and correlates with the adult rostrum length. Moreover, in long-beaked dolphins such as *Stenella attenuatai*, the premaxillary cartilage is still present at the very tip of the rostrum at Carnegie stage 23 [117], which is longer than in the mouse, where the premaxilla has already ossified by this stage (approx. E15.5) [110]. Mechanistically, cartilage elongation might be achieved by the additional formation of new adjacent mesenchymal condensations, extended phase of chondrocyte proliferation or by delayed onset of the nasal cartilage ossification [24,25,79].

The transcription factor Runx2 controls the commitment of craniofacial mesenchyme to osteogenic lineage and regulates the proliferation of osteoblast progenitors and the maturation of hypertrophic chondrocytes [118]. During the evolution of eutherian mammals, Runx2 extreme transactivation potential emerged, and correlates with the poly-glutamine and poly-alanine (QA) tandem repeat length and the Q : A residue ratio [119]. Runx2 protein activity (i.e. transactivation potential) correlates with species-specific facial length variation in carnivores, primates and bats [120–123]. Higher protein activity is associated with longer facial proportions in carnivores and primates, while in bats the higher activity levels correlate with wider and shorter facial proportions [120]. Interestingly, marsupials have conserved QA repeat length despite possessing facial length diversity comparable to placentals [124]. This indicates that the Runx2 protein structure represents only one of the mechanisms generating facial length variation.

The shape and size of craniofacial bones are Runx2 dose-dependent in mice and humans. Decreased levels lead to brachycephaly and hypertelorism [125,126], while the increased dose of Runx2 causes premature cranial vault suture closure (i.e. craniosynostosis) [127]. Moreover, changes in the regulation of *Runx2* expression during human evolution were linked to skull globularization (i.e. more rounded, less elongated skull) and a delay in cranial suture closure in modern humans [128]. Runx2 activity levels and the resulting heterochrony in suture closure may account for higher postnatal brain growth rates in early hominids, facilitating encephalization (i.e. increase in brain volume relative to body size) [129].

Skull globularization and encephalization are mammalian innovations associated with the evolution of mammalian-specific brain region, namely the neocortex, and with the expansion of the olfactory cortex and cerebellum [130,131]. The increase in brain volume is associated with cranial vault expansion [132,133] and changes in ossification timing [30]. It has been proposed that the morphological differences between Neanderthal and modern human skulls are due to differences in brain growth rates and the onset of ossification during development [134,135]. Heterochrony in the onset of ossification of the skull and suture closure is widely observed across mammals and is associated with skull shape variation [30,136]. Early onset of cranial vault ossification, particularly in the bones protecting the neocortex, olfactory cortex and cerebellum, correlates with the level of encephalization across mammals [30].

Heterochrony in cranial suture closure has been mainly studied in the context of craniosynostosis, a pathological condition manifested by the premature closure of the cranial sutures [137]. The cranial sutures are formed by soft tissue separating the craniofacial bones, enabling postnatal bone growth and regulating cranial vault expansion [138,139]. Besides brain growth retardation and cognitive dysfunction, craniosynostosis also causes craniofacial dysmorphisms [140]. Evidence from patients with craniosynostosis shows that suture closure patterns produce consistent abnormal skull shapes [141,142]. Interestingly, the characteristic skull shape in brachycephalic dogs is also associated with a particular timing of cranial suture closure [143]. These observations support that the timing of cranial suture closure represents an important source of skull shape variability. In nature, levels of cranial suture closure (i.e. the number of closed sutures in adulthood) vary significantly among mammals and are correlated to foraging styles [136,144,145]. However, as feeding ecology is tightly linked to morphological diversification, the timing of the suture closure might represent the mechanistic link between these two traits.

### (e) Mammalian dentition

Among the mammalian innovations is the presence of tooth classes (incisors, canines, premolars and molars), also known as heterodonty, and diphyodont dentition (i.e. the presence of two sets of teeth, one deciduous, another permanent) [146,147]. Teeth formation relies upon interactions between the ectomesenchyme and the oral ectoderm, particularly the dental lamina [148]. Contrary to polyphyodonts (i.e. the majority of toothed fishes and reptiles) that constantly grow and replace teeth throughout their lives, the dental lamina of diphyodont animals degrades after the formation of the second set of teeth during embryonic development [147,149]. This event correlates with the downregulation of canonical WNT signalling in the successional (replacement) dental lamina (i.e. the dental lamina that generates the second tooth set) [150]. Tooth replacement also requires the specific expression pattern of the following genes: *Sox2* in the primary and replacement dental lamina, *Sostdc1* (WNT signalling antagonist) between the deciduous tooth germs and the replacement dental lamina, and *Runx2* in the tooth mesenchyme [149–151]. Downregulation of *Sox2* and *Runx2* and activation of WNT signalling in the dental epithelium leads to the production of supernumerary teeth associated with the expansion of the dental lamina [151–153]. The heterochronic persistence of the dental lamina is observed in non-mammalian polyphyodonts [154,155] and could represent the underlying cause of the extremely rare cases of polyphyodonty in mammals [156,157].

Mice posses one set of teeth that is not replaced throughout their life, thus, they are monophyodont mammals [158,159]. The absence of a second set of teeth in mice has been linked to the lack of canonical WNT signalling in the rudimentary replacement lamina [159]. Mice also possess continuously growing incisors (hyperdontia), a capacity linked to the expression of *Sox2* in the dental epithelial stem cells located in the cervical loop of the incisors [151,160]. *Sox2*-expressing dental stem cells in the cervical loop are also observed in the permanent premolars and molars of the rabbit, which, similar to the mouse incisors, are ever-growing teeth [150]. On the contrary, in the mouse molars and deciduous premolars and molars of rabbits, which do not constantly grow, *Sox2* expression is reduced compared to the ever-growing teeth [150,151,160]. The post-canine teeth of aardvarks, sloths and some armadillos are also ever-growing [161]; thus, such heterochronic shift and persisting *Sox2* expression in the epithelial dental stem cells may be the underlying cause of the permanent teeth growth in these mammals.

Mammalian heterodonty is associated with regionalized gene expression along the proximo-distal axis of the jaws. For instance, in possums and ferrets, *Msx1/Barx1* overlapping expression in the jaw mesenchyme marks the premolar, while *Barx1* expression is restricted to the molar primordium [162,163]. Contrary to this, in the mouse model, the *Msx1/Barx1* expression overlap is minimal [162], corresponding to the lack of premolars but the presence of molars in these animals. The expression of *Msx1/Barx1* is controlled by the regionalized expression of *Bmp4* and *Fgf8*, which are typically restricted to the caudal and rostral regions of the oral epithelium, respectively [164–167]. Moreover, the *Fgf8* expression pattern is associated with multicuspid teeth such as molars, while *Bmp4* is associated with unicuspid teeth like incisors [167–169]. Interestingly, *Fgf8* spatial expression in dolphins extends into the caudal part of the jaws and overlaps with the *Bmp4* expression domain [170]. The heterotopy of *Fgf8* and *Bmp4* expression has been proposed to underlie the characteristic unicuspid (homodont) incisor-like dentition observed in these cetaceans. Similar unicuspid supernumerary dentition is also present in phocid seals and giant armadillos [171,172]. Thus, it may be possible that the dolphin-like expression pattern of *Fgf8* and *Bmp4* underlies the teeth patterning and morphogenesis in these species as well.

## 4. Conclusion

To acquire a mechanistic understanding of the origin of skull shape variation, it is essential to recognize the interconnection between individual steps of head development and gain a more profound knowledge of species-specific differences on the genetic, genomic, molecular, cellular and tissue levels. Similar to other vertebrates, craniofacial shape variation in mammals arises primarily during embryogenesis. However, while comparative vertebrate embryology studies have been carried out extensively in the last decades, a systematic study within the mammalian lineage has not yet been performed. Until recently, the possibility of acquiring a holistic understanding of developmental process divergence across mammals was out of reach due to technological limitations. However, the increasing availability of whole-genome annotations across mammals enables us to select species based on their genome homology or divergence for comparative studies.

For instance, genetically similar species with different skull morphologies will ultimately allow us to grasp how genetic information is integrated to orchestrate developmental mechanisms and generate a morphological variation. Further comparative investigation of individual developmental steps will allow identifying the causative modulation that altered the subsequent morphogenetic events.

Recent technological advances such as single-cell omics and related bioinformatic analyses reveal cell type representation, cellular origin, the sequence of cell fate decisions and the molecular signature of each cell. Even minor molecular gear differences may significantly impact future cell fate decisions and affect the consecutive developmental steps. Therefore, employing single-cell omics along an extended developmental timeline could pinpoint the origin of developmental divergence between species and allow us to comprehend the nature and extent of subsequent changes leading to specific skull shapes. Modern bioinformatic tools allow the integration of single-cell multi-omics data with genomic analyses and link the differential gene expression dynamics to the regulatory landscape. The necessary validations of cluster identities that emerged from the bioinformatic analysis of the single-cell data are also substantially easier due to the possibility to visualize individual mRNA molecules using species-specific probes (such as single-molecule *in situ* hybridization chain reaction, HCR). Ultimately, tissue contrasting and micro-computed tomography enable the assessment of the developing skull morphology before ossification and allow to focus on the relevant embryonic stages.

Contrary to these advancements, obtaining developmental series across mammalian species still represents a bottleneck. Evolutionary developmental biology often relies on museum collections to obtain specimens for three-dimensional imaging and morphometrics, and complete developmental series of non-model species are relatively rare. Furthermore, considering that mammalian development occurs *in utero*, the research shall be carefully designed to minimize the number of animals used and comply with the 3R principles (replacement, reduction and refinement) of animal welfare. However, mammals represent an enormously informative model for comparative investigations of the developmental process divergence and will allow linking the effects of heterochrony, heterometry, heterotopy and other developmental modulators to species-specific molecular and cellular patterns and processes.

## Data Availability

This article has no additional data.
